# A delta-radiomic lymph node model using dynamic contrast enhanced MRI for the early prediction of axillary response after neoadjuvant chemotherapy in breast cancer patients

**DOI:** 10.1186/s12885-022-10496-5

**Published:** 2023-01-05

**Authors:** Shasha Liu, Siyao Du, Si Gao, Yuee Teng, Feng Jin, Lina Zhang

**Affiliations:** 1 Department of Radiology, The First Hospital of China Medical University, Shenyang, 110001 China; 2 Departments of Medical Oncology and Thoracic Surgery, The First Hospital of China Medical University, Shenyang, 110001 China; 3 Department of Breast Surgery, The First Hospital of China Medical University, Shenyang, 110001 China

**Keywords:** Breast neoplasms, Axillary lymph node, DCE-MRI, Radiomics, Neoadjuvant chemotherapy, Pathological complete response

## Abstract

**Background:**

The objective of this paper is to explore the value of a delta-radiomic model of the axillary lymph node (ALN) using dynamic contrast-enhanced (DCE) MRI for early prediction of the axillary pathological complete response (pCR) of breast cancer patients after neoadjuvant chemotherapy (NAC).

**Methods:**

A total of 120 patients with ALN-positive breast cancer who underwent breast MRI before and after the first cycle of NAC between October 2018 and May 2021 were prospectively included in this study. Patients were divided into a training (*n* = 84) and validation (*n* = 36) cohort based on the temporal order of their treatments. Radiomic features were extracted from the largest slice of targeted ALN on DCE-MRI at pretreatment and after one cycle of NAC, and their changes (delta-) were calculated and recorded. Logistic regression was then applied to build radiomic models using the pretreatment (pre-), first-cycle(1st-), and changes (delta-) radiomic features separately. A clinical model was also built and combined with the radiomic models. The models were evaluated by discrimination, calibration, and clinical application and compared using DeLong test.

**Results:**

Among the three radiomic models, the ALN delta-radiomic model performed the best with AUCs of 0.851 (95% CI: 0.770–0.932) and 0.822 (95% CI: 0.685–0.958) in the training and validation cohorts, respectively. The clinical model yielded moderate AUCs of 0.742 (95% CI: 0.637–0.846) and 0.723 (95% CI: 0.550–0.896), respectively. After combining clinical features to the delta-radiomics model, the efficacy of the combined model (AUC = 0.932) in the training cohort was significantly higher than that of both the delta-radiomic model (Delong *p* = 0.017) and the clinical model (Delong *p* < 0.001) individually. Additionally, in the validation cohort, the combined model had the highest AUC (0.859) of any of the models we tested although this was not statistically different from any other individual model’s validation AUC. Calibration and decision curves showed a good agreement and a high clinical benefit for the combined model.

**Conclusion:**

This preliminary study indicates that ALN-based delta-radiomic model combined with clinical features is a promising strategy for the early prediction of downstaging ALN status after NAC. Future axillary MRI applications need to be further explored.

**Supplementary Information:**

The online version contains supplementary material available at 10.1186/s12885-022-10496-5.

## Introduction

Neoadjuvant chemotherapy (NAC) is now widely used in clinically node-positive breast cancer patients to allow for more limited surgery in the breast and axilla [1]. Approximately 35–68% of positive axillary lymph nodes (ALN) before treatment go on to achieve axillary pathologic complete response (pCR) after NAC [2], thus specialists might want to omit axillary lymph node dissection (ALND) to avoid the related complications such as limited shoulder mobility and upper arm lymphedema [3–5]. Sentinel lymph node biopsy (SLNB) has become the standard of treatment for breast cancer patients with clinically negative lymph nodes [6]. However, as a result of tumor burden, lymphatic fibrosis after chemotherapy, and nonuniform tumor regression of metastatic ALNs [7], the accuracy of SLNB is unsatisfactory, and this limits its widespread use in ycN0 patients. Furthermore, false-negative rates for sentinel lymph node (SLN) can be in excess of 10% [8, 9] for women who present with clinically positive axilla but downstage to clinically negative axilla. Therefore, there is an urgent need to discover reliable, noninvasive biomarkers capable of identifying patients whose ALNs are expected to respond completely after NAC.

The field of radiomics can be used to mine high-throughput quantitative and noninvasive image features to improve cancer diagnosis and treatment, and has attracted much attention in recent years. Radiomics based on analysis of breast MRI has already shown satisfactory prediction accuracy in benign and malignant differentiation [10], molecular typing [11], and treatment response prediction [12, 13]. Additionally, radiomic models of primary tumor and ALN on DCE-MRI have been demonstrated to be capable of predicting ALN metastasis preoperatively [14, 15] and of determining ALN status after NAC [16]. Delta-radiomics is an emerging field in cancer efficacy assessment that can provide an estimation of the change in tumor heterogeneity and aggressiveness before and after cancer therapy [17]. This radiomics subfield has been found to have the ability to predict the response to chemotherapy of many primary tumors in many types of cancer [18, 19], including primary breast cancer [13, 20]. However, the question of whether delta-radiomic features of the lymph nodes themselves can help to predict early axillary response after NAC has not been investigated.

In this paper, we studied whether the radiomic changes of ALN were able to reflect the early response of ALNs accurately and if they could be used as a biomarker for axillary pCR prediction after NAC. To do this, we built one clinical model and three radiomic models based on MRI features from baseline, after one cycle of treatment, and based on the changes between these two points (delta-radiomic features). These three ALN-based radiomic models were compared, and a clinical-radiomic combined model was developed for the purpose of early prediction of axillary pCR after NAC.

## Materials and methods

The prospective protocol of this study was approved by the Scientific Research Ethics Committee of the First Hospital of China Medical University, and each participant provided written informed consent.

### Study population and NAC protocol

We identified 158 consecutive patients treated with NAC followed by surgery between October, 2018 and May, 2021. The inclusion criteria were: (1) biopsy-proven ipsilateral metastatic ALNs with locally advanced breast cancer; (2) availability of the complete biopsy information of the primary tumor; and (3) standard breast MRI conducted both before and after one cycle of NAC. A total of 38 patients were excluded for the following reasons: (1) small ALN diameter (< 1.0 cm) on baseline MRI (*n* = 17); (2) artifacts in the axillary region on DCE-MRI (*n* = 9); (3) incomplete standard NAC cycles (*n* = 2); (4) occurrence of distant metastases during NAC (*n* = 4); (5) unfinished surgery (*n* = 2); and (6) surgery performed at other institutions (*n* = 4). The final patient population (*n* = 120) was organized in its original temporal order and divided (7:3) into a training cohort (*n* = 84) for model development and a validation cohort (*n* = 36) for model validation.

The NAC regimen administered to all patients was according to the National Comprehensive Cancer Network (NCCN) guidelines [21]. The NAC regimens in our institution (Table S2) were described as follows: six cycles of the TEC regimen (docetaxel, epirubicin and cyclophosphamide); four cycles of the EC regimen with sequential four cycles of docetaxel (total 8 cycles) for HER2-negative tumors. For HER2-positive tumors, dual anti-HER2-targeted trastuzumab plus pertuzumab or single trastuzumab were added to the chemotherapy drugs, including TCbHP (docetaxel, carboplatin, trastuzumab and pertuzumab), and TCH (docetaxel, cyclophosphamide and trastuzumab). Each regimen was administered intravenously every 3 weeks. All patients underwent breast-conserving surgery or total mastectomy after NAC. For axillary management, all patients underwent ALND after NAC.

### Histopathological analysis

Clinical information was collected including patient age, menopausal status, estrogen receptor (ER) and progesterone receptor (PR), human epidermal growth factor receptor-2 (HER2) status, Ki-67, molecular subtypes, pretreatment clinical T/N stage, posttreatment pathologic T/N stage (ypTN). ER, PR and HER2 were evaluated according to ASCO/USCAP guidelines [22, 23]. The Ki-67 index was assessed with a cut-off value of 20% [24]. The molecular subtype was classified into luminal A, luminal B, HER2-enriched and triple negative according to the 2017 St. Gallen guidelines [25]. Clinical and pathologic tumor stage was assessed according to the American Joint Committee on Cancer TNM staging system manual, 8th edition [26]. After NAC, axillary pCR was defined as the absence of any invasive residual cancer in the axillary region [26]. Breast pCR was defined as the absence of residual invasive tumor (Miller–Payne grade 5, residual ductal carcinoma in situ could be present) [12].

### Image acquisition

The baseline MRI was performed within 1 week prior to NAC, and the follow-up MRI was performed after the first cycle of NAC (within 72 h before the second cycle of NAC). Both breast MRI examinations were performed with a 3 T MR scanner (SIGNATM Pioneer, GE Healthcare, Milwaukee, WI, USA) with an 8-channel phased-array breast coil. The patients were positioned prone with both breasts naturally draped over the middle of the coil. A multi-phase ultra-fast contrast enhancement technology, which was called Differential Subsampling with Cartesian Ordering (DISCO), was used to create the T1-weighted DCE-MRI including one pre-contrast and 20 post-contrasts (GE Healthcare). After the pre-contrast scan, contrast medium (gadodiamide, 0.1 mmol/kg body weight, GE Healthcare, Ireland) was injected via intravenous route with a power injector, followed by a 20-ml saline flush. The DCE-MRI sequence parameters were as follows: TR = 4.9 ms, TE = 1.7 ms, field of view (FOV) = 360 × 360 mm, matrix = 256 × 256, section thickness = 5 mm, intersection gap = 1.3 mm, number of sections = 120/phase, and acceleration factor = 2. The scan sequences and scan parameters are shown in Table S1.

### Analysis workflow

**Fig. 1:**
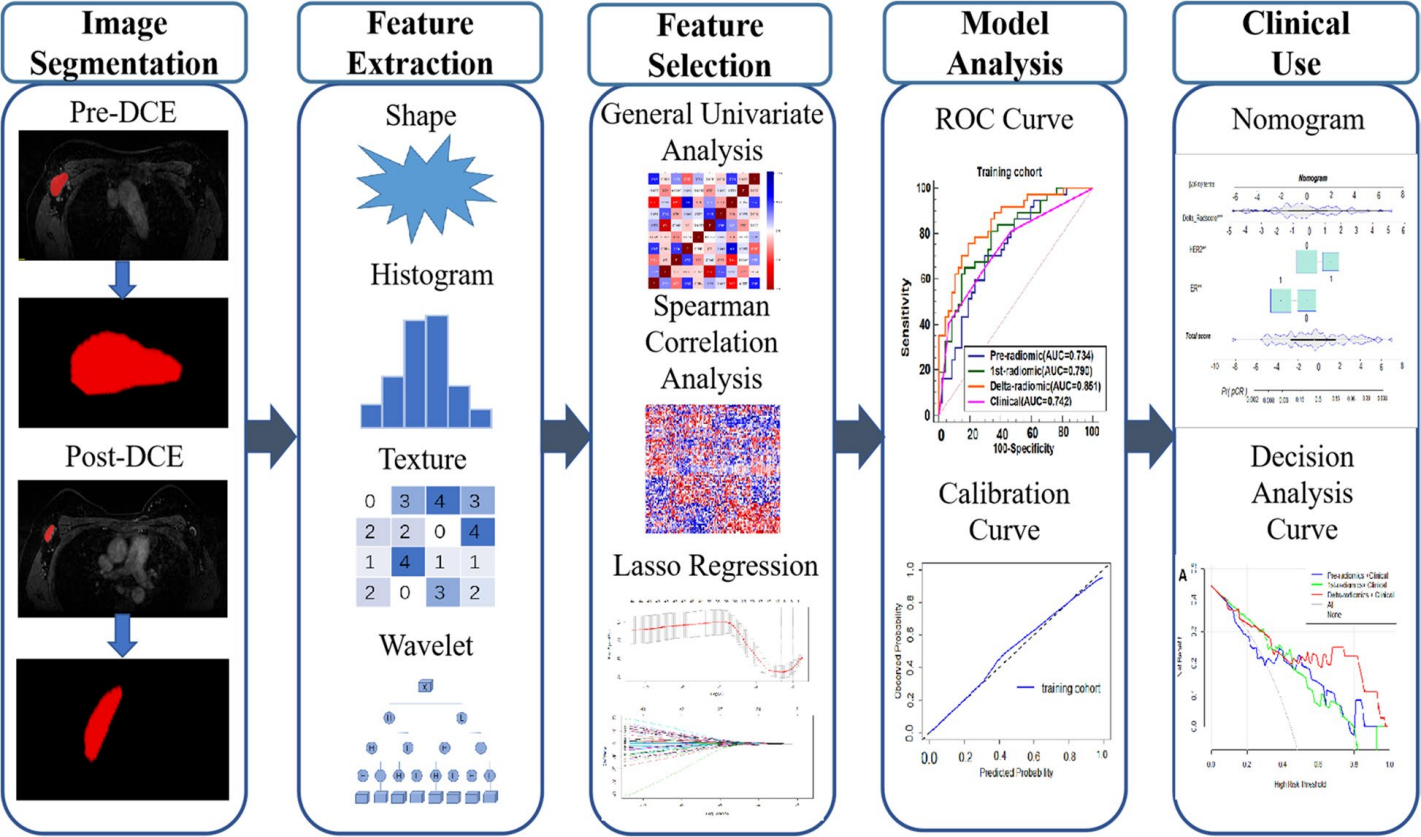
The workflow of the critical steps. DCE dynamic contrast enhanced, LASSO least absolute shrinkage and selection operator, ROC receiver operating characteristic

The prediction workflow consisted of identifying the ALN region of interest (ROI) segmentation, radiomic feature extraction, feature selection, model building, and then model evaluation (Fig. 1).

### Ipsilateral ALN segmentation

**Fig. 2:**
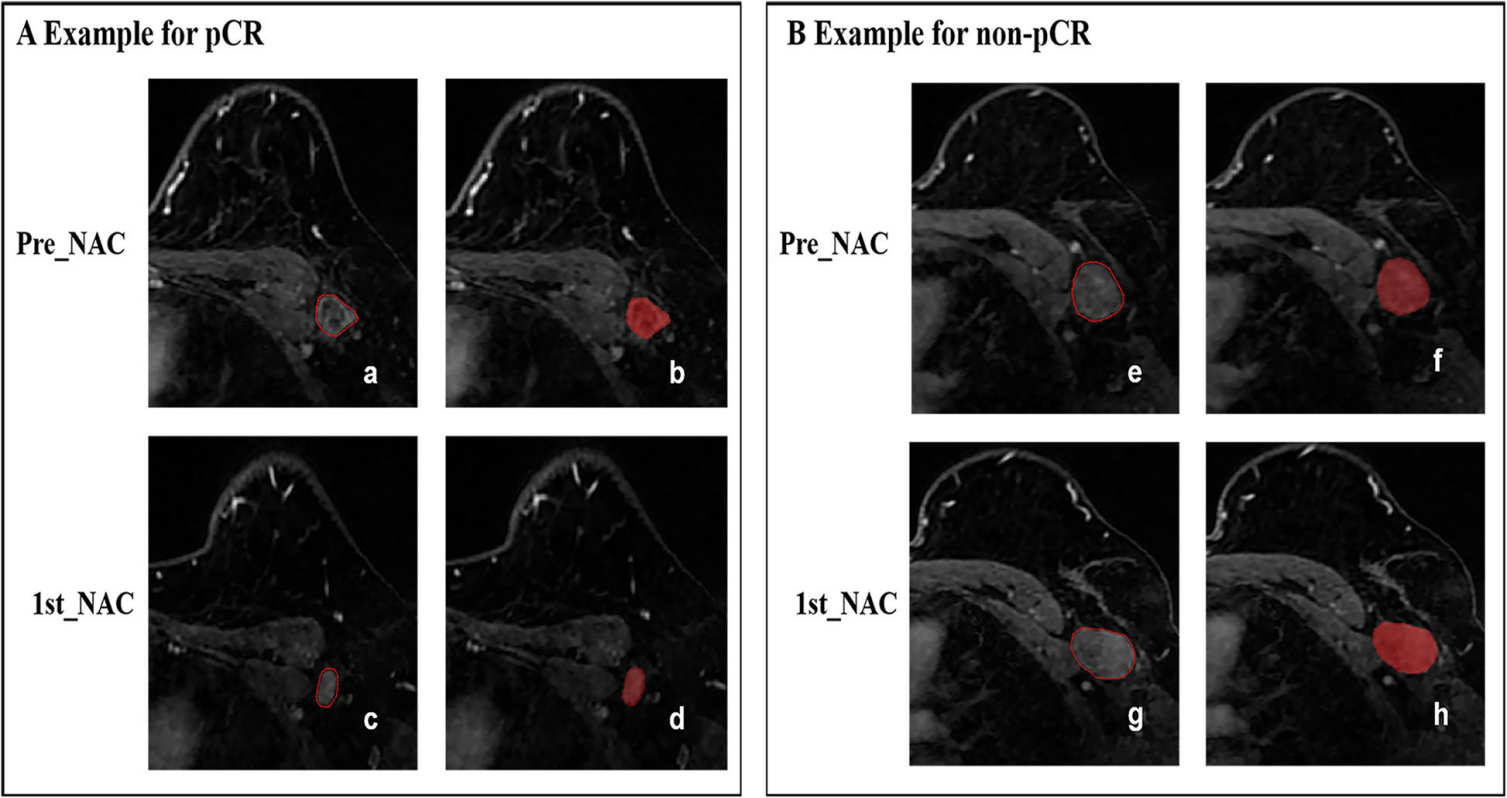
Representative images segmentation. **A** Images from an axillary pCR breast cancer patient (aged 34 years old with invasive ductal carcinoma of metastasis in the left ALNs). **B** Images from an axillary non-pCR breast cancer patient (aged 55 years old with invasive ductal carcinoma of metastasis in the left ALNs). a-b and e-f were the baseline DCE-MRI; c-d and g-h were the first cycle DCE- MRI). ALN axillary Lymph node, DCE dynamic contrast enhanced, apCR axillary pathologic complete response

On the baseline and follow-up CE-MRI, the 2D region of interest (ROI) at the maximum cross-sectional area of one selected ALN was manually segmented on the peak contrast phase according to the DCE curve (obtained during 116–136 s after contrast injection) by using the open-source ITK-snap software (www.itksnap.org, version 3.8.0). Typical features of selected ALN on DCE-MRI were: cortical thickening, loss of fatty hilum, and a round shape or a long-to-short axis ratio of less than 2 [27, 28]. If multiple enlarged lymph nodes were available, the lymph node with the longest short diameter according to the biopsy record was selected as the targeted region of interest. The segmentations for all cases were performed by one radiologist with 5 years of experience in breast imaging, and then 30 randomly selected cases were segmented again by another radiologist with 10 years of experience in breast imaging. Consistently, the long and short diameter of selected positive ALN before and after one cycle of NAC was also measured by the same radiologists. Both radiologists were blind to the clinical and histopathological data. The reliability of the observations was calculated using the intraclass correlation coefficient (ICC). Features with ICCs greater than 0.75 indicated satisfactory reproducibility and were reserved for further analysis. The representative DCE-MRI images before and after one cycle of NAC are shown in Fig. 2.

### Radiomic features and their changes

According the 2D-ROI of the ALNs, 851 separate radiomic features before and after the first cycle were extracted from the peak phase of DCE-MRI using Analysis Kit software (A.K., GE Healthcare). The extracted features included: original features (*n* = 107), first-order statistics (*n* = 18), shape-based features (*n* = 14), the gray level co-occurrence matrix (GLCM, *n* = 24), the gray level run length matrix (GLRLM, *n* = 16), the gray level size zone matrix (GLSZM, *n* = 16), the neighboring gray tone difference matrix (NGTDM, *n* = 5), the gray level dependence matrix (GLDM, *n* = 14), and wavelet-transformed type (*n* = 744). Changes in the radiomic features (delta-radiomic features) were calculated from the differences between the pre-treatment features values (pre-radiomic features) and the feature values after one cycle of NAC (1st-radiomic features):

$$\textrm{Delta}-\textrm{radiomic}\ \textrm{features}=\left(\textrm{Pre}-\textrm{radiomic}\ \textrm{features}\right)-\left(1\textrm{st}-\textrm{radiomic}\ \textrm{features}\right).$$

The change in the long and short diameter of ALN after treatment was also calculated using the same formula.

### Feature selection

In the training cohort, the radiomic features were normalized to a Z-score to make the dynamic ranges comparable before radiomic feature selection. Spearman correlation analysis was then used to remove the features that were highly correlated with the other features using a cutoff value for |r| of 0.9. To obtain the features that were most strongly associated with axillary pCR, we performed univariate regressions analysis and features with *p* < 0.1 were selected for subsequent analysis. Finally, we used the least absolute shrinkage and selection operator (LASSO) for fine feature selection. The tuning parameter (λ) was selected by 5-fold cross-validation, and the value of λ was then adjusted to minimize the binomial deviation of the model, which was the point at which the efficiency of the filtered features is optimal [29].

### Building the model

The prediction models were developed by the multivariable regression with the Akaike’s information criterion (AIC) in training cohort. The prediction Radiomic scores (Rad-score) was calculated for each patient using the linear fusion of the selected non-zero features and their coefficients. Our radiomics models included pre-, 1st-, and delta-radiomic versions, that were constructed with the radiomic features from the baseline MRI, the MRI after one cycle of NAC, and from the changes between these two images, respectively. The clinical model was built by combining the independent predictors among all clinical factors that were found by stepwise multivariate logistic regression. Finally, clinical features were combined with each of the three radiomic models in order to establish combined models: the pre-radiomic + clinical model, the 1st-radiomic + clinical model, and the delta-radiomic + clinical model. To help us discern the optimal prediction model with the highest performance, we developed an individualized nomogram to provide a visual tool for evaluating the prediction of the patients’ ALN pCR.

### Model evaluation and validation

The performance of the prediction models was evaluated using receiver operating characteristic (ROC) analysis and was also compared with the DeLong test in both the training and validation cohorts. The area under the curve (AUC) with 95% confidence interval (CI), sensitivity, specificity, and accuracy were also calculated to assess model performance. The clinical utility of the models was determined and compared using decision curve analysis (DCA) to quantify the net benefit to the patients under different threshold probabilities in training and validation cohorts. The consistency between the expected probability of axillary pCR and the actual results was shown using calibration curves for the nomogram.

### Statistical analysis

Clinicopathological characteristics were compared between the two groups by using the Mann-Whitney U test for continuous variables and the chi-square test or Fisher exact test for categorical variables. Statistical analysis was performed using Med Calc (version 15.6.1) and R software (version 3.6.1; http://www.r-project.org/). For all statistical tests, we considered two-sided *p* < 0.05 to indicate statistically significant test results.

## Results

### Patient’s characteristics and clinical model

**Fig. 3:**
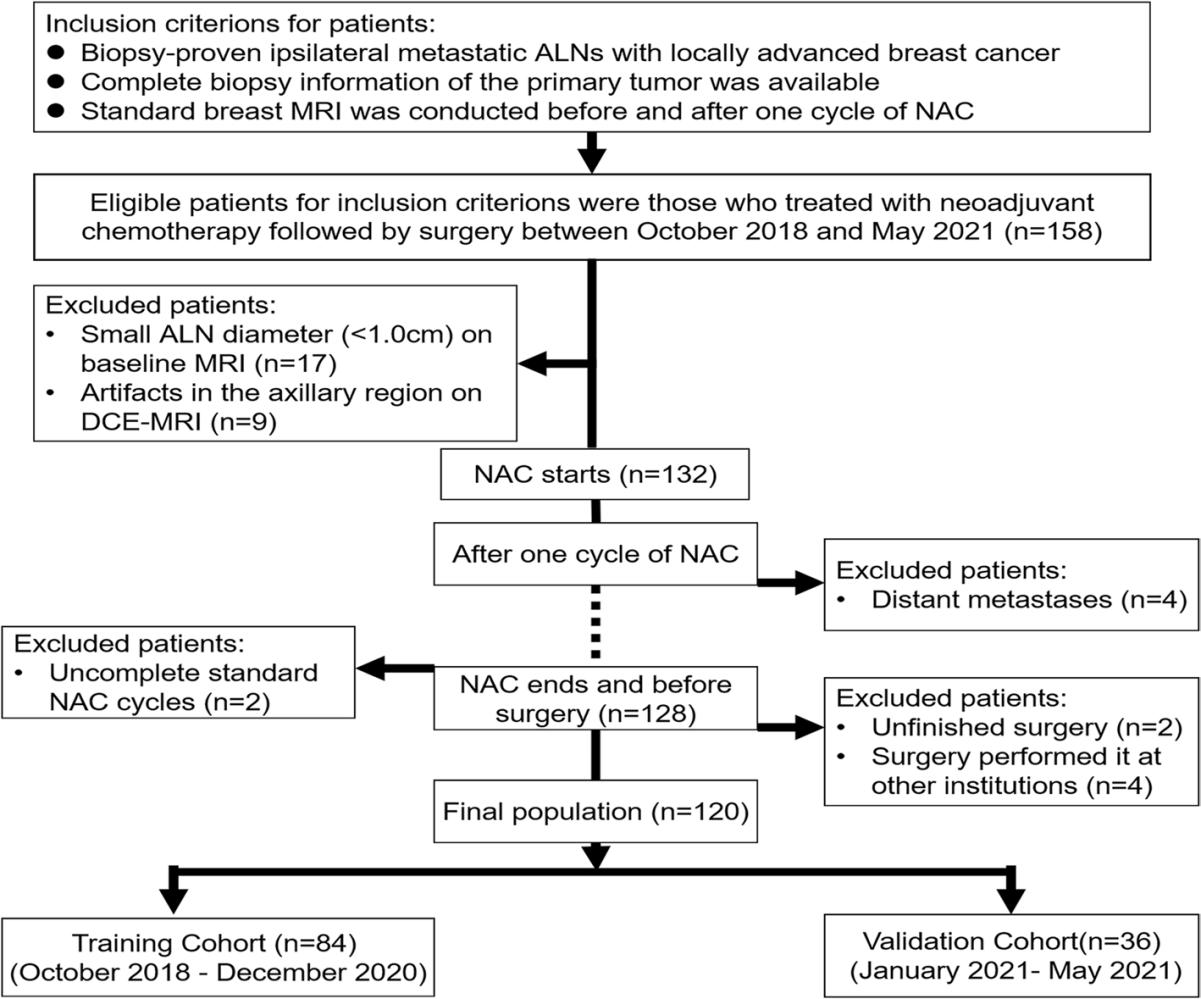
Flowchart of patient recruitment pathway. ALN axillary Lymph nodes, NAC neoadjuvant chemotherapy, DCE dynamic contrast enhanced

One hundred twenty women comprised the final study group (mean age ± standard deviation, 50.9 years ±10.1; range, 31–73 years), The training cohort included 84 cases and the validation cohort included 36 cases. The agreement between the two radiologists based on nodule size measurements was good, with an ICC range of 0.94–0.99. Flowchart of patient enrollment is shown in Fig. 3 and final patients’ characteristics are listed in Table 1.

**Table 1:** Baseline characteristics for both cohorts

Characteristics	Training cohort (*n* = 84)			Validation cohort (*n* = 36)		
	apCR (*n* = 37)	non-apCR (*n* = 47)	*P*	apCR (*n* = 16)	non-apCR (*n* = 20)	*p*
Age (years)	50.5 ± 8.7	50.7 ± 10.9	0.928	49.8 ± 11.5	53.3 ± 9.6	0.329
Menopausal (%)			0.679			1.000
Premenopausal	18 (48.6)	25 (53.2)		7 (43.8)	8 (40.0)	
Postmenopausal	19 (51.4)	22 (46.8)		9 (56.2)	12 (60.0)	
ER status (%)			0.001			0.191
Positive	13 (35.1)	33 (70.2)		5 (31.2)	11 (55.0)	
Negative	24 (64.9)	14 (29.8)		11 (68.8)	9 (45.0)	
PR status (%)			0.022			0.500
Positive	16 (43.2)	32 (68.1)		8 (50.0)	13 (65.0)	
Negative	21 (56.8)	15 (31.9)		8 (50.0)	7 (35.0)	
HER2 status (%)			0.002			0.023
Positive	21 (56.8)	11 (23.4)		12 (75.0)	7 (35.0)	
Negative	16 (43.2)	36 (76.6)		4 (25.0)	13 (65.0)	
Ki-67 status (%)			0.659			0.764
≤ 20%	5 (13.5)	8 (17.0)		3 (18.8)	3 (15.0)	
> 20%	32 (86.5)	39 (83.0)		13 (81.2)	17 (85.0)	
Molecular subtypes (%)			0.005			0.073
Luminal A	1 (2.7)	5 (10.6)		0 (0.0)	1 (5.0)	
Luminal B	17 (45.9)	29 (61.7)		8 (50.0)	13 (65.0)	
HER2 enriched	12 (32.4)	2 (4.3)		6 (37.5)	1 (5.0)	
TN	7 (18.9)	11 (23.4)		2 (12.5)	5 (25.0)	
Clinical T stage (%)			0.993			0.525
T1	6 (16.2)	8 (17.0)		2 (12.5)	5 (25.0)	
T2	15 (40.5)	20 (42.6)		10 (62.5)	8 (40.0)	
T3	8 (21.6)	9 (19.1)		1 (6.2)	3 (15.0)	
T4	8 (21.6)	10 (21.3)		3 (18.8)	4 (20.0)	
Clinical N stage (%)			0.317			0.273
N1	26 (70.3)	32 (68.1)		14 (87.5)	12 (60.0)	
N2	9 (24.3)	8 (17.0)		1 (6.2)	4 (20.0)	
N3	2 (5.4)	7 (14.9)		1 (6.2)	4 (20.0)	
Pre-LD (mm)	17.2 ± 6.9	18.3 ± 5.8	0.456	16.2 ± 4.9	18.9 ± 6.5	0.183
1st-LD (mm)	12.9 ± 5.2	14.5 ± 5.2	0.171	12.4 ± 4.7	14.8 ± 4.1	0.108
Pre-SD (mm)	12.9 ± 3.6	14.1 ± 4.6	0.184	12.8 ± 3.5	13.6 ± 4.1	0.547
1st-SD (mm)	9.7 ± 3.3	11.1 ± 4.6	0.130	9.4 ± 3.9	10.6 ± 2.9	0.294
Delta-LD (mm)	4.3 ± 2.5	3.8 ± 3.3	0.418	3.8 ± 2.4	4.0 ± 4.5	0.851
Delta-SD (mm)	3.2 ± 1.3	3.0 ± 2.8	0.769	3.4 ± 2.1	3.0 ± 3.4	0.665
Breast pCR (%)						
Yes	17 (45.9)	5 (10.6)	< 0.001	13 (81.2)	2 (10.0)	< 0.001
No	20 (54.1)	42 (89.4)		3 (18.8)	18 (90.0)	

Age is presented as mean ± SD, and others shown as proportions (percentages)

*ER* Estrogen receptor, *PR* Progesterone receptor, *HER2* Human epidermal growth factor receptor 2, *TN* Triple negative, *apCR* Axillary pathologic complete response, pre- pretreatment, *1st* One cycle, *LD* Long diameter, *SD* Short diameter, *pCR* Pathologic complete response

Axillary pCR was observed in 53 (44.2%) cases (training/validation, *n* = 37/16). The pCR of primary breast tumor was observed in 37 (30.8%) cases (training/validation, *n* = 22/15).The axillary pCR rate was significantly higher than that of the breast (*p* < 0.001). The pCR concordance rate in axilla and breast was 25% (30/120). In patients with axillary pCR, only 56.5% (30/53) achieved breast pCR; While in breast pCR, nearly 81.2% (30/37) achieved axillary pCR (Fig. S1).

The ER, PR, and HER2 expression, molecular subtypes and breast pCR were significantly different between the axillary pCR group and non-pCR group in the training cohort (*p* < 0.05), and HER2 expression and breast pCR were statistically different in the validation cohort (*p* < 0.05) (Table 1). We found no statistical difference in any of the clinical characteristics between the training and validation cohorts (*p* > 0.05) (Table S2).

The ER, PR, and HER2 expression and molecular subtype (*p* < 0.05) in the training cohort were initially included to build the clinical model, and the stepwise method mentioned above preserved only ER and HER2 expression as independent predictors in the final clinical model (Table 2). This final clinical model yielded an AUC of 0.742 (95% CI: 0.637–0.846) in the training cohort, and 0.723 (95% CI: 0.550–0.896) in the validation cohort for predicting axillary pCR.

**Table 2:** Multivariate analysis used stepwise based on logistic regression predicting apCR in training cohort

Characteristics	OR	95%CI	*P*
ER status (%)			
Positive	1		
Negative	3.982	1.515–10.466	0.005
HER2 status (%)			
Positive	1		
Negative	0.256	0.095–0.687	0.007

*ER* Estrogen receptor, *HER2* Human epidermal growth factor receptor 2, *apCR* Axillary pathologic complete response

### Radiomic models for axillary pCR prediction

**Fig. 4:**
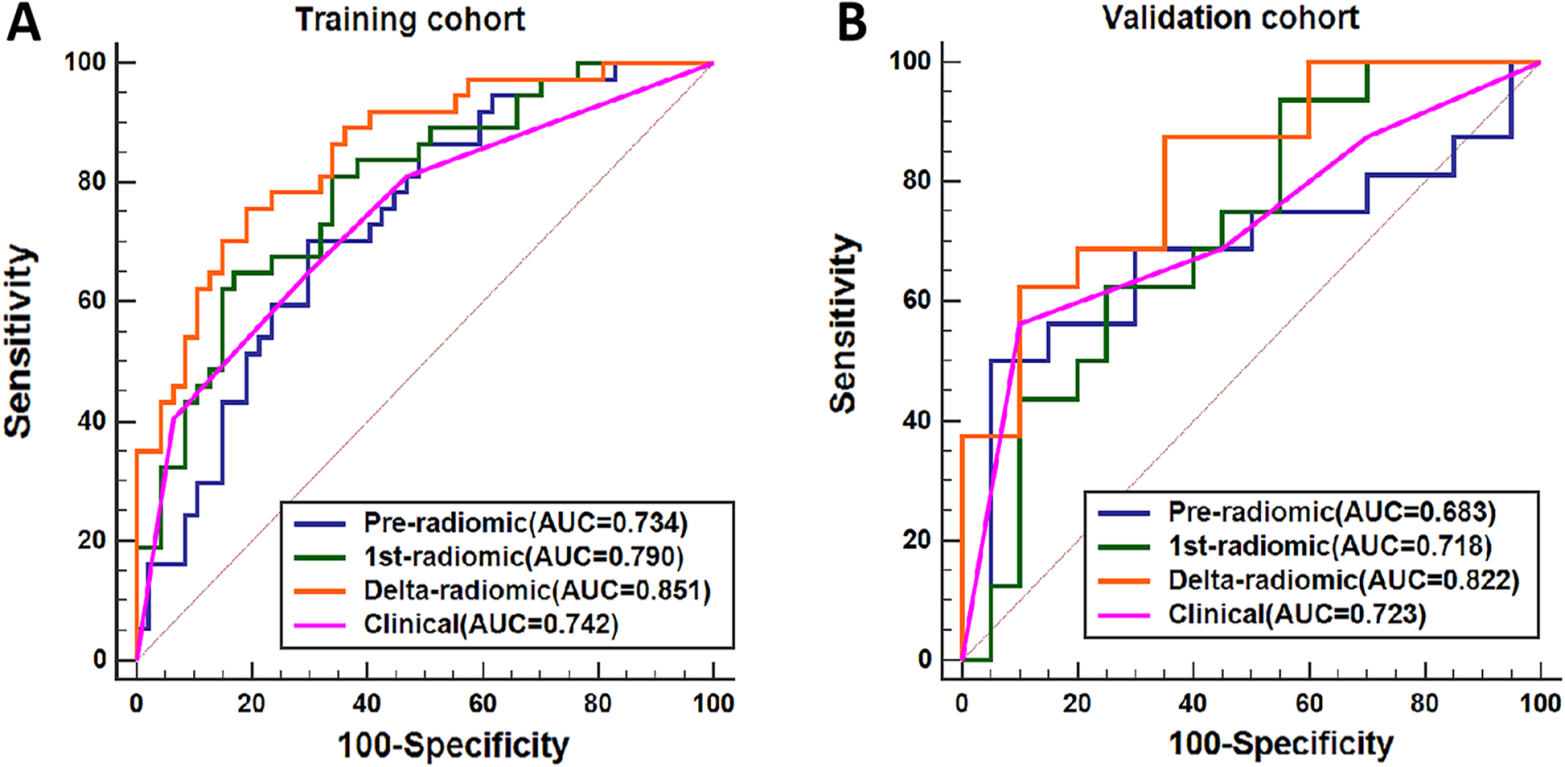
The ROC curves of the separate models for predicting apCR. **A** Training cohort. **B** Validation cohort. apCR axillary pathologic complete response

The ICCs for all radiomic features and their changes were greater than 0.75 between the two radiologists. Thus, all extracted features were included in the subsequent analysis. The pre-, 1st-, and delta-radiomic models were eventually selected for two, three, and four features for predicting axillary pCR, respectively. The final-selected key radiomic features of the three radiomic models and their equations are listed in Table S3. The delta-radiomic model had the highest AUC for predicting axillary pCR after NAC: 0.851 (95% CI: 0.770–0.932) in the training cohort and 0.822 (95% CI: 0.685–0.958) in the validation cohort. Figure 4 shows the ROC curves of the three radiomic models in the training (Fig. 4A) and validation (Fig. 4B) cohorts, and Table 3 details the models’ predictive performance.

**Table 3:** The performance of the radiomic models in the training cohort and validation cohort

Models	Training cohort				Validation cohort			
	SEN	SPE	ACC	AUC (95% CI)	SEN	SPE	ACC	AUC (95% CI)
Pre-radiomic	0.703	0.702	0.702	0.734 (0.628–0.841)	0.500	0.950	0.750	0.683 (0.486–0.876)
1st-radiomic	0.648	0.829	0.750	0.790 (0.693–0.886)	0.937	0.450	0.666	0.718 (0.548–0.888)
Delta-radiomic	0.757	0.809	0.786	0.851 (0.770–0.932)	0.875	0.650	0.750	0.822 (0.685–0.958)
Clinical	0.648	0.702	0.678	0.742 (0.637–0.846)	0.562	0.900	0.750	0.723 (0.550–0.896)
Pre-radiomic +Clinical	0.837	0.702	0.761	0.829 (0.734–0.915)	0.625	0.900	0.778	0.734 (0.549–0.918)
1st-radiomic+ Clinical	0.703	0.936	0.833	0.831 (0.739–0.922)	0.750	0.800	0.778	0.809 (0.667–0.951)
Delta-radiomic + Clinical	0.865	0.894	0.881	0.932 (0.882–0.983)	0.750	0.850	0.806	0.859 (0.733–0.985)

*AUC* Area under the ROC, *95%CI* 95% confidence interval, *SEN* Sensitivity, *SPE* Specificity, *ACC* Accuracy

### Clinical-radiomic models for axillary pCR prediction

**Fig. 5:**
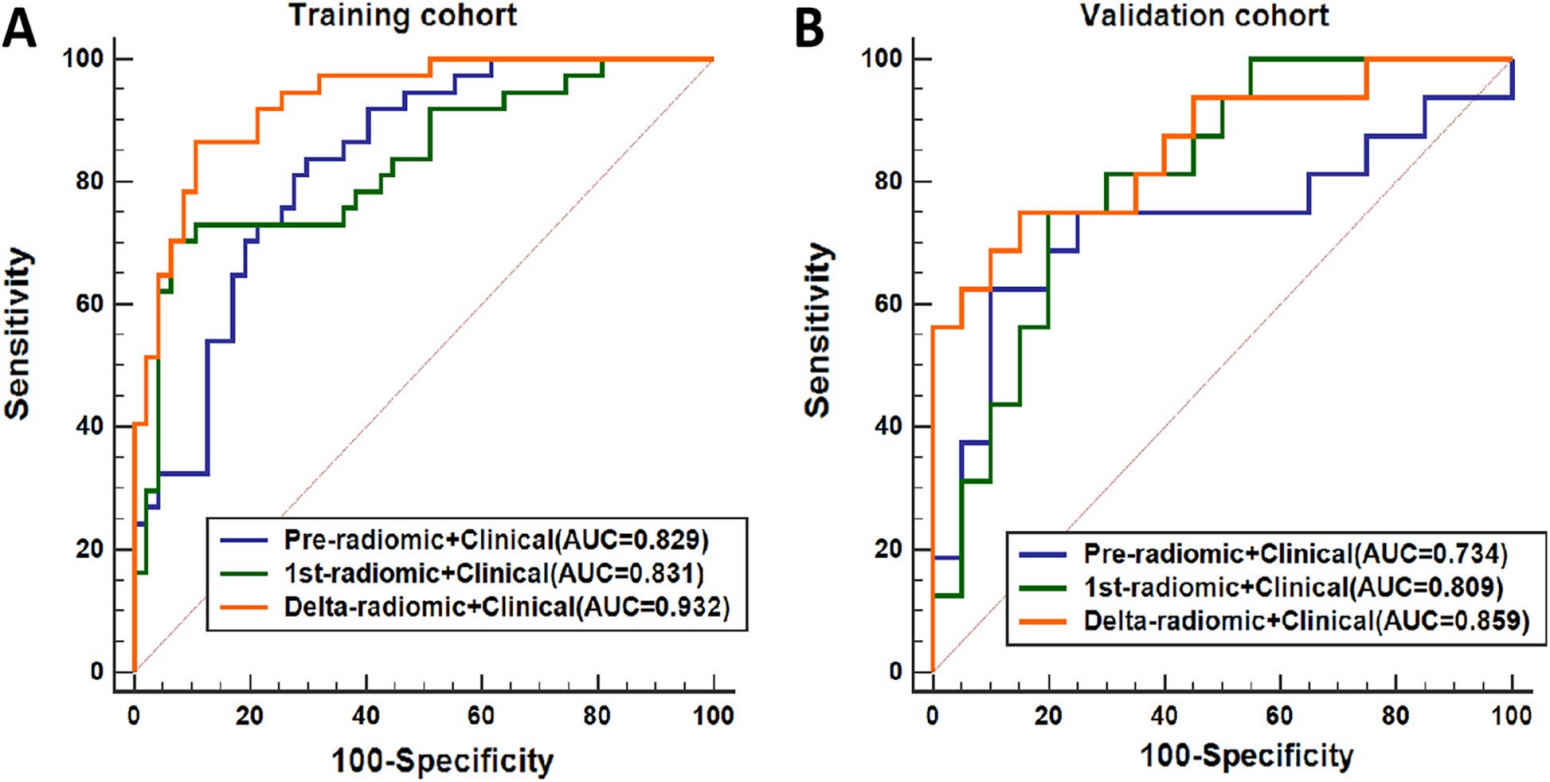
The ROC curves of the combined models for predicting apCR. **A** Training cohort. **B** Validation cohort. apCR axillary pathologic complete response

Figure 5 shows the ROC analysis results of the three combined clinical-radiomic models (pre-radiomic + clinical, 1st-radiomic + clinical, and delta-radiomic + clinical) in the training (Fig. 5A) and validation (Fig. 5B) cohorts.

**Fig. 6:**
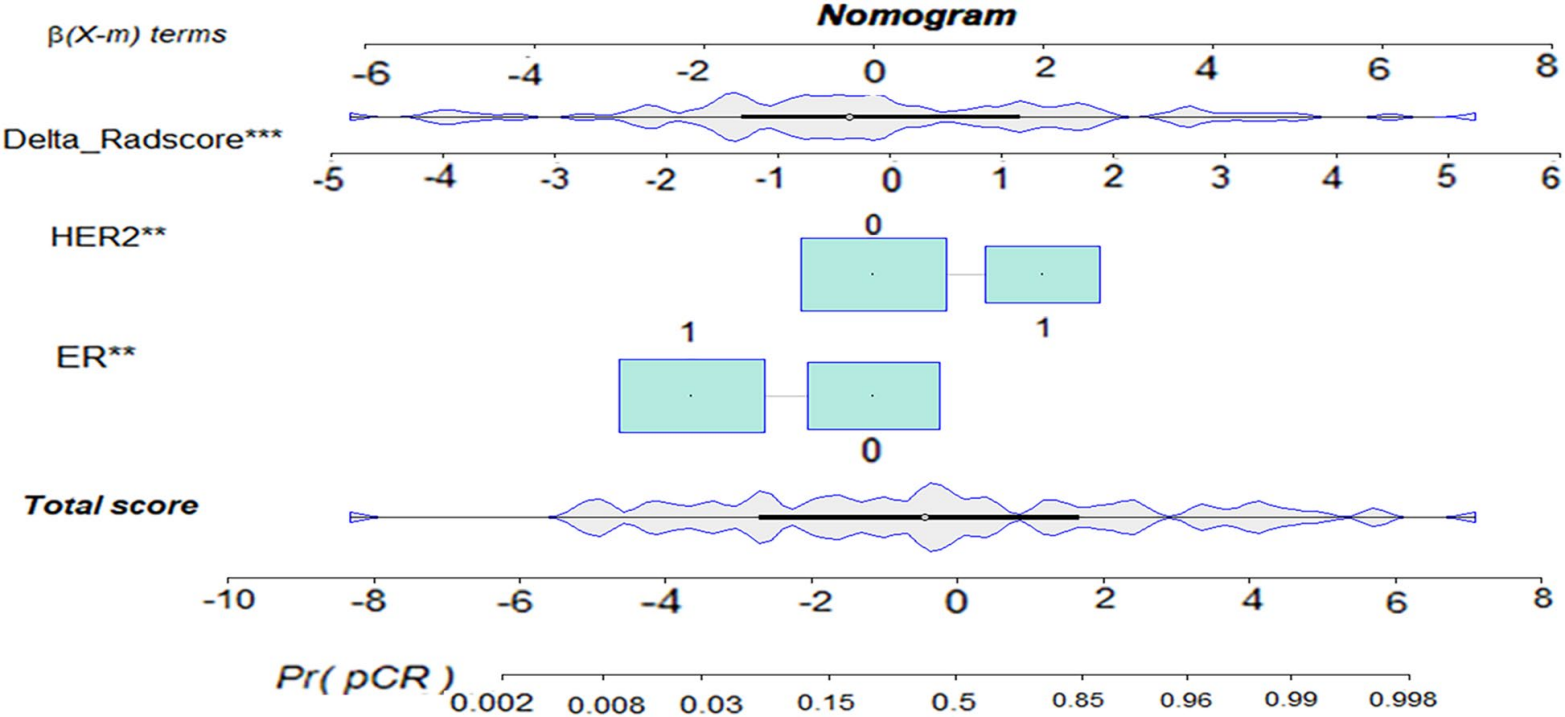
Visual nomogram of the delta-radiomic + clinical model in predicting apCR. The ** represents. *p* value < 0.01, *** represents. *p* value < 0.001, apCR axillary pathologic complete response

The delta-radiomic + clinical model yielded the highest AUC of these models: 0.932 (95% CI: 0.882–0.983) in the training cohort and 0.859 (95% CI: 0.733–0.985) in the validation cohort. We developed an individualized nomogram for visualization (Fig. 6).

**Fig. 7:**
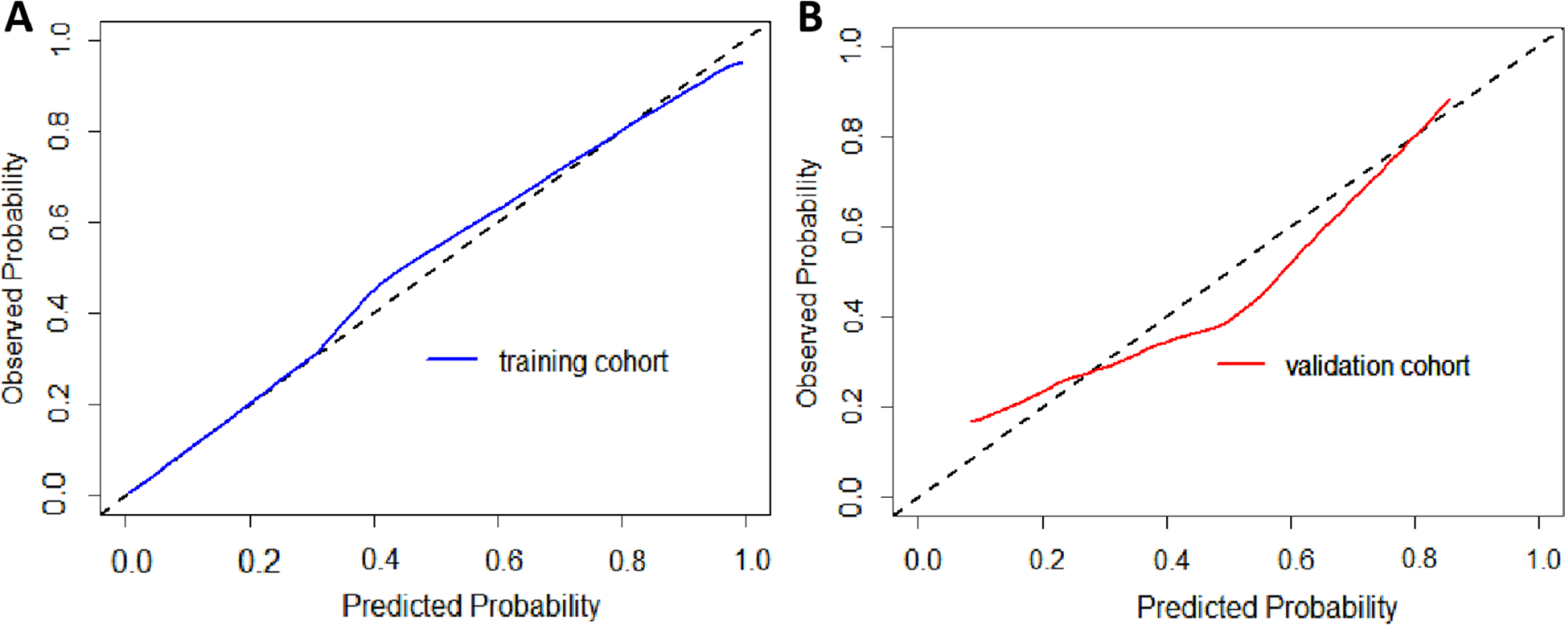
Calibration curves for nomogram. **A** Training cohort. **B** validation cohort. The X-axis represents the predicted probability of apCR estimated by nomogram, whereas the Y-axis represents the actual apCR rates. Calibration curves show that the actual probability corresponded closely to the prediction of nomogram. apCR axillary pathologic complete response

The calibration curves showed agreement between the predictions and actual observations of the nomogram in both the training (Fig. 7A) and validation cohorts (Fig. 7B).

**Fig. 8:**
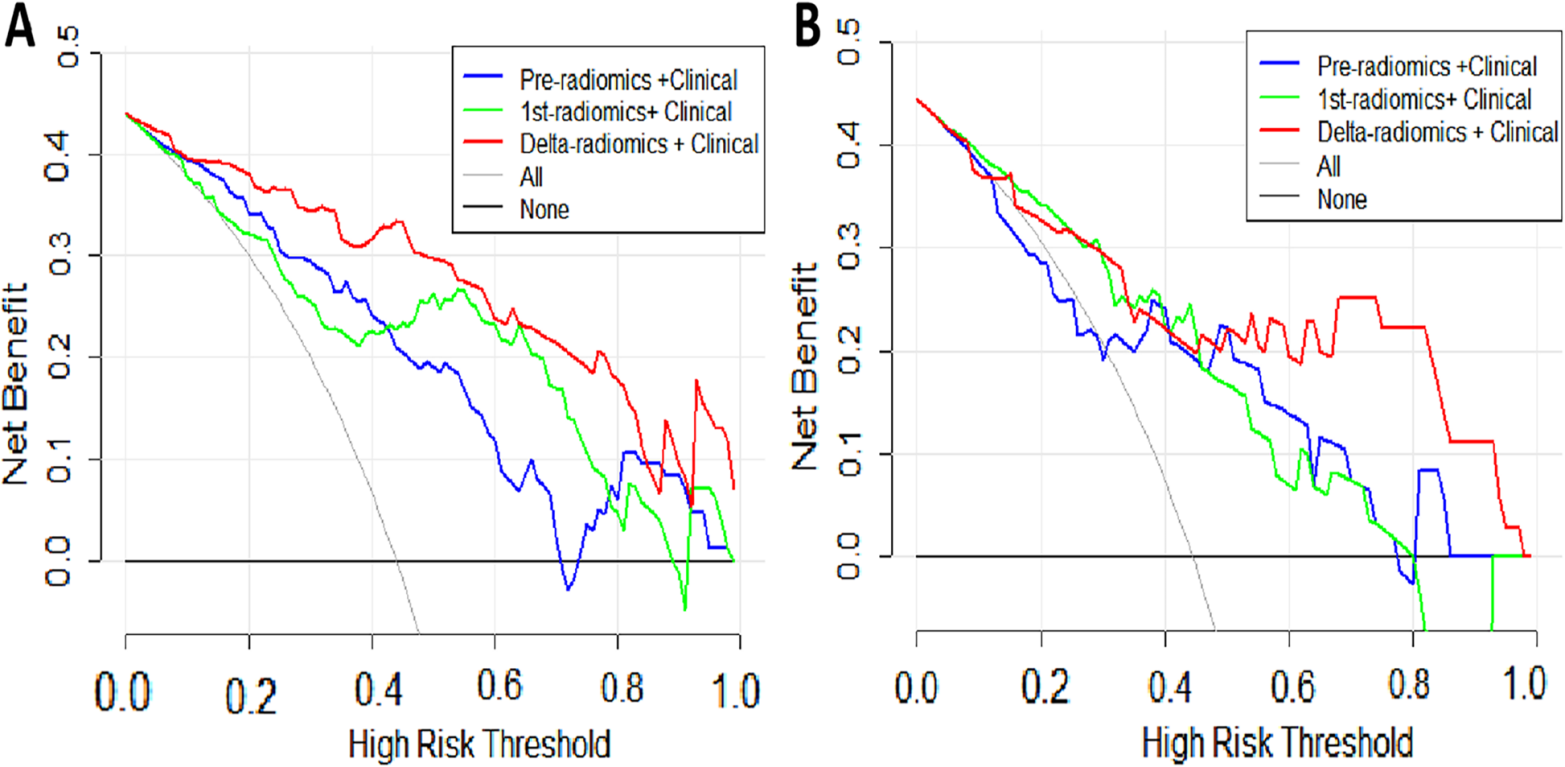
Decision curve analysis (DCA) of the combined models. **A** Training cohort. **B** validation cohort. The x-axis indicates the threshold probability, while the y-axis indicates the net benefit. The gray line indicates the hypothesis that all the patients achieved an apCR, and the black line indicates the hypothesis that none of the patients achieved an apCR. apCR axillary pathologic complete response

Additionally, our DCA indicated that the clinical benefit of the combined delta-radiomic + clinical model was greater than both the pre-radiomic + clinical and 1st-radiomic + clinical models in distinguishing axillary pCR when the threshold probability was between 0 and 0.85 in the training cohort (Fig. 8A), and when the threshold probability was between 0.53 and 0.98 in the validation cohort (Fig. 8B).

### Model comparison

The predictive efficiency of the delta-radiomic + clinical model was higher than that of the delta-radiomic, clinical, pre-radiomic + clinical, and 1st-radiomic + clinical models (Delong test: *p* = 0.017; *p* < 0.001; *p* = 0.002; *p* = 0.007) in the training cohort. Comparisons of the following models were also statistically different: pre-radiomic + clinical vs. pre-radiomics/clinical, 1st-radiomic + clinical vs. clinical, with *p* < 0.05 for all DeLong tests. In the validation cohort, the combined delta-radiomic + clinical model had the highest AUC (0.859) and accuracy (0.806). However, these values were not statistically different from those of any other model at the 0.05 level (Fig. S2).

## Discussion

We investigated the performance of ALN-related radiomic models for axillary pCR prediction at baseline and after early-treatment and also investigate the changes between these two time-points. The results indicated that the delta-radiomic model based on early changes of ALN features performed better among all radiomic models. Moreover, when combined with clinical features, the ALN delta-radiomic + clinical model achieved the best diagnostic performance of any model we tested. The delta-radiomic model that incorporates clinical and ALN-MRI features may be a promising method for ALN pCR prediction in the initial phase of NAC and for further treatment decisions.

Of the three radiomic models we constructed, the ALN delta-radiomic model showed the highest predictive value. Intratumor heterogeneity drives neoplastic progression and therapeutic response [30, 31] and changes dynamically accompanied by size changes after treatment [30]. Delta-radiomics can show the heterogeneity of changing information, which is ignored by single time-point models [18, 19]. Fan et al. [13] reported that the performance of a delta-radiomic model after two cycles of NAC exceed the baseline model for pCR prediction based on primary breast cancer, and Gan et al. [16] found that a preoperative radiomic model from visible ALNs had higher predictive power compared with a model based on the MRI features of breast tumor and axillary region. This result implicates the importance of introducing ALN features for breast cancer model building. Our study advanced the predictive time-point to one-cycle treatment and presented the early changes of ALN radiomic features associated with treatment response after NAC. Considering pCR inconsistency between the breast and axilla, the development of specialized axillary pCR prediction models after NAC could complement the breast pCR prediction, rather than incorporate of breast/axilla in the definition of pCR. The treatment adjustment according to the model’s prediction depends mainly on the breast response, while an additional axillary non-pCR on the basis of breast non-pCR will enhance the clinician’s confidence for NAC regimen change. For our delta-radiomic model, the first-cycle may be the only feasible time-point: treatment-driven ALN shrinkage makes it difficult to identify and obtain reliable radiomic features.

Receptor status, Ki-67 level, and breast pCR could all influence axillary pCR, with moderate predictive ability (AUC 0.715 to 0.804) [32–34], and our model built using ER and HER2 was similar to this. Breast pCR was not used to establish clinical models due to its delayed availability after surgery, rather than early treatment. According to the “seed and soil” hypothesis, metastasis is the product of interactions between selected cancer cells (the seeds) and specific organ microenvironments (the soil) [35]. However, the clinical features of the primary tumor cannot completely replace the ALN itself. A previous study [14] proposed that tumor radiomic signatures or a combination of tumor and ALN radiomic signatures were no better than ALN radiomic signatures alone for preoperative ALN metastasis prediction. In our study, we used radiomic features of ALNs alone to predict axillary pCR after NAC, and achieved satisfactory performance. Furthermore, we used regular breast MRI involving axillary level I without entire axilla scans. Although regular MRI protocol limits the visualization of high-level ALNs, it is sufficient to exclude high-level and advanced ALN metastasis [36]. Both regular MRI protocol and dedicated axillary MRI have been shown to have comparable performance in ALN metastasis evaluation [37].

Compared to the primary breast tumor, visible ALNs have simple physiological structure and small variation between patients, which is conducive to ROI delineation. For this reason and to simplify our workflow, we applied time-saving 2D single-slice delineation instead of whole-slice delineation in this study. Single-slice analysis has often been considered to have the same diagnostic ability as 3D whole-slice analysis [12, 38]. To a certain extent, this also reduces the impact of axillary artifacts caused by poor field uniformity on radiomic features. Thus, in this paper we selected the most suspicious metastatic ALN to represent all the ALNs. Clinicians can easily find the corresponding ALN after early-treatment according to its original location.

The use of MRI for axilla assessment is becoming more popular in clinic due to its advantage of a more global view of the axilla that can enhance the detection of potentially abnormal ALNs and allow the comparison of both axillary irrespective of patient body habitus [39]. Future axillary MRI applications still need to be explored, however, including the development of dedicated and stable axillary coils, the necessity of high-level axillary scan, the value of MRI morphology, quantification, and radiomics. In the current study, we used regular breast MRI to build radiomic models based on positive and visible ALNs. We mention that this result will lay the foundation for future radiomics studies with dedicated axillary MRI.

In the setting of invasive breast cancer with positive ALNs, our ALN-radiomic model can help to determine the presence of residual LN metastases at the initial stage of NAC and may even increase confidence in intended treatment plans or help patients and providers to decide among multiple available treatment modalities. As the trend toward less-aggressive axillary surgery continues, a more precise yet encompassing role for imaging might be required in axillary evaluation. Providing more accurate post-treatment response evaluation can help to minimize intervention and optimize patient outcomes.

The present study is not without its limitations, however. First, the sample size was relatively small, but larger cohorts are being recruited for future deep learning analysis. Second, NAC regimens were not uniform and sequential regimens were also used, such as EC-T. This might affect the accuracy of delta-radiomic model based on the first cycle treatment to some extent. Third, molecular subtype-specific subgroup analysis could not be completed due to limited patient numbers within each subgroup. HER2-positive and triple-negative subgroup analyses might yield higher predictive efficacy due to their higher axillary pCR rate [40]. Then, radiomic features from primary breast tumors were not combined with those of ALNs. However, Gan et al. [16] suggested that nodal features alone are sufficient for residual axillary cancer prediction. Finally, the inherent limitations of the methodology, such as using a single ALN, and single-slice ROI, will be addressed in our future study involving a large-scale axillary DCE-MRI.

In conclusion, this preliminary study indicates that our ALN-based delta-radiomics model combined with clinical features is a promising strategy for the early prediction of the downstaging of ALN status after NAC.

## Supplementary Information

**Additional file 1: Table S1.** Details of sequence information and scan parameters. **Table S2.** Clinicopathologic characteristics of patients in the training and validation cohorts. **Table S3.** Key features for each signature and the formulas of optimal radiomic score. **Figure S1.** Lymph node and breast pathologic complete response (pCR) are illustrated. **Figure S2.** Delong test for all models in the training and validation cohorts.

## Data Availability

The data presented in this study are available on reasonable request from the corresponding author LZ, zhanglnda@163.com. Due to privacy restrictions the data are not publicly available.

## Abbreviations

ALN: Axillary lymph node
AUC: Area under curve
DCE-MRI: Dynamic contrast-enhanced magnetic resonance imaging
NAC: Neoadjuvant chemotherapy
PCR: Pathological complete response
ROC: Receiver operating characteristic
ROI: Region of interest
ALND: Axillary lymph node dissection
SLNB: Sentinel lymph node biopsy
SLN: Sentinel lymph node
NCCN: National Comprehensive Cancer Network
DISCO: Differential Subsampling with Cartesian Ordering
ICC: Intraclass correlation coefficient
ER: Estrogen receptor
PR: Progesterone receptor
HER2: Human epidermal growth factor receptor-2
GLCM: Gray level co-occurrence matrix
GLRLM: Gray level run length matrix
GLSZM: Gray level size zone matrix
NGTDM: Neighbouring gray tone difference matrix
GLDM: Gray level dependence matrix
LASSO: Least absolute shrinkage and selection operator
DCA: Decision curve analysis
