# Supplementary material for: A delta-radiomic lymph node model using dynamic contrast enhanced MRI for the early prediction of axillary response after neoadjuvant chemotherapy in breast cancer patients
Source: BMC Cancer. 2023 Jan 5;23:15. doi: 10.1186/s12885-022-10496-5 (PMC9817310; doi:10.1186/s12885-022-10496-5)
Supplement: Supplementary file 1 — Additional file 1: Table S1. Details of sequence information and scan parameters. Table S2. Clinicopathologic characteristics of patients in the training and validation cohorts. Table S3. Key features for each signature and the formulas of optimal radiomic score. Figure S1. Lymph node and breast pathologic complete response (pCR) are illustrated. Figure S2. Delong test for all models in the training and validation cohorts. [file 12885_2022_10496_MOESM1_ESM.docx]

**Supplementary Material**

**Table S1** Details of sequence information and scan parameters.

| Parameters | T1WI | T2WI | DWI* | DCE |
| --- | --- | --- | --- | --- |
| Scan plane | Axial | Axial | Axial | Axial |
| Imaging technique | 2D FSE | 2D FSE | EPI | 3D DISCO |
| Echo time (ms) | 6.3 | 84.9 | 65.5 | 1.7 |
| Repetition time (ms) | 498 | 4975 | 2535 | 4.9 |
| Fat suppression | No | Yes | Yes | Yes |
| Field of view (mm) | 360×360 | 360×360 | 360×360 | 360×360 |
| Matrix | 320×256 | 320×256 | 128×160 | 256×256 |
| Section thickness (mm) | 5 | 5 | 5 | 1.4 |
| No. of sections | 25 | 25 | 25 | 120/phase |
| Acceleration factors | 2.5 | 2.5 | 2 | 2 |
| Acquisition time (min: s) | 1:40 | 1.59 | 2.32 | 6.48(1+20 phases) |

The initial located imaging lasted 29 seconds, thus the total scan time was 13:28 min; *b=0,50,400,800s/mm2; DWI = diffusion-weighted imaging; FSE = fast spin echo; EPI = echo planar imaging; DISCO = differential subsampling with cartesian ordering; DCE = dynamic contrast-enhanced.

**Table S2** Clinicopathologic characteristics of patients in the training and validation cohorts.

| Characteristics | Total cohort  (120) | Training cohort  (n=84) | Validation cohort  (n=36) | *P* |
| --- | --- | --- | --- | --- |
| Age (years) | 50.9±10.1 | 50.6±10.0 | 51.8±10.5 | 0.561 |
| Menopausal (%) |  |  |  | 0.339 |
| Premenopausal | 58(48.3) | 43(51.2) | 15(41.7) |  |
| Postmenopausal | 62(51.7) | 41(48.8) | 21(58.3) |  |
| ER status (%) |  |  |  | 0.300 |
| Positive | 62(51.7) | 46(54.8) | 16(44.4) |  |
| Negative | 58(48.3) | 38(45.2) | 20(55.6) |  |
| PR status (%) |  |  |  | 0.904 |
| Positive | 69(57.5) | 48(57.1) | 21(58.3) |  |
| Negative | 51(42.5) | 36(42.9) | 15(41.7) |  |
| HER2 status (%) |  |  |  | 0.136 |
| Positive | 51(42.5) | 32(38.1) | 19(52.8) |  |
| Negative | 69(57.5) | 52(61.9) | 17(47.2) |  |
| Ki-67 status (%) |  |  |  | 0.870 |
| ≤20% | 19(15.8) | 13(15.5) | 6(16.7) |  |
| >20% | 101(84.2) | 71(84.5) | 30(83.3) |  |
| Molecular subtypes (%) |  |  |  | 0.792 |
| Luminal A | 7(5.8) | 6(7.1) | 1(2.8) |  |
| Luminal B | 67(55.8) | 46(54.8) | 21(58.3) |  |
| HER2 enriched | 21(17.5) | 14(16.7) | 7(19.4) |  |
| TN | 25(20.8) | 18(21.4) | 7(19.4) |  |
| NAC regimen |  |  |  |  |
| TEC | 49(40.8) | 36(42.9) | 13(36.1) | 0.471 |
| EC-T | 20(16.7) | 16(19.0) | 4(11.1) |  |
| TCbHP | 29(24.2) | 18(21.4) | 11(30.6) |  |
| TCH | 22(18.3) | 14(16.7) | 8(22.2) |  |
| Clinical T stage (%) |  |  |  | 0.625 |
| T1 | 21(17.5) | 14(16.7) | 7(19.4) |  |
| T2 | 53(44.2) | 35(41.7) | 18(50.0) |  |
| T3 | 21(17.5) | 17(20.2) | 4(11.1) |  |
| T4 | 25(20.8) | 18(21.4) | 7(19.4) |  |
| Clinical N stage (%) |  |  |  | 0.668 |
| N1 | 84(70.0) | 58(69.0) | 26(72.2) |  |
| N2 | 22(18.3) | 17(20.2) | 5(13.9) |  |
| N3 | 14(11.7) | 9(10.7) | 5(13.9) |  |
| ypT stage (%) |  |  |  | 0.234 |
| T0 | 37(30.8) | 22(26.2) | 15(41.7) |  |
| T1 | 28(23.3) | 19(22.6) | 9(25.0) |  |
| T2 | 41(34.2) | 34(40.5) | 7(19.4) |  |
| T3 | 9(7.5) | 6(7.1) | 3(8.3) |  |
| T4 | 5(4.2) | 3(3.6) | 2(5.6) |  |
| ypN stage (%) |  |  |  | 0.992 |
| N0 | 53(44.2) | 37(44.0) | 16(44.4) |  |
| N1 | 42(35.0) | 29(34.5) | 13(36.1) |  |
| N2 | 15(12.5) | 11(13.1) | 4(11.1) |  |
| N3 | 10(8.3) | 7(8.3) | 3(8.3) |  |
| Pre-LD (mm) | 17.8 ± 6.2 | 17.8 ± 6.3 | 17.7± 5.9 | 0.916 |
| 1st-LD (mm) | 13.8 ± 5.0 | 13.8 ± 5.2 | 13.7 ± 4.5 | 0.940 |
| Pre-SD (mm) | 13.5 ± 4.1 | 13.6 ± 4.2 | 13.2 ± 3.8 | 0.662 |
| 1st-SD (mm) | 10.4 ± 3.9 | 10.5 ± 4.1 | 10.1 ± 3.4 | 0.620 |
| Delta-LD (mm) | 4.0 ± 3.2 | 4.0 ± 3.0 | 3.9 ± 3.7 | 0.931 |
| Delta-SD (mm) | 3.1 ± 2.4 | 3.1 ± 2.2 | 3.1 ± 2.9 | 0.948 |
| Breast pCR (%) |  |  |  |  |
| Yes | 37(30.8) | 22(26.2) | 15(41.7) | 0.093 |
| No | 83(69.2) | 62(73.8) | 21(58.3) |  |

Age is presented as mean ± SD, and others shown as percentage. ER, estrogen receptor; PR, progesterone receptor; HER2, human epidermal growth factor receptor 2; TN, triple negative; TEC, docetaxel, epirubicin and cyclophosphamide; EC-T, epirubicin, cyclophosphamide and docetaxel; TCbHP, docetaxel, carboplatin trastuzumab and pertuzumab; TCH, docetaxel, cyclophosphamide and trastuzumab; pre-, pretreatment; 1st-, one cycle; LD, long diameter; SD, short diameter; pCR, pathologic complete response

**Table S3** Key features for each signature and the formulas of optimal radiomic score.

| Signatures | Key features | Formula |
| --- | --- | --- |
| Pre-radiomic | Wavelet-HHH_gldm_DependenceEntropy  wavelet-LHH_gldm_  SmallDependenceLowGrayLevelEmphasis | Pre-radiomic=-0.42906+1.03342*wavelet-HHH_gldm_DependenceEntropy+  0.96387*wavelet-LHH_gldm_ SmallDependenceLowGrayLevelEmphasis |
| 1st-radiomic | original_ngtdm_Coarseness  wavelet-HHH_glcm_Idmn  wavelet-LLH_glcm_ClusterProminence | 1st-radiomic= 0.33925+0.61230*original_ngtdm_Coarseness  +0.56437*wavelet-HHH_glcm_Idmn+1.24074*wavelet-LLH_glcm_ClusterProminence |
| Delta-radiomic | original_firstorder_Skewness  Wavelet-HHL_glszm_GrayLevelNonUniformity  wavelet-LLH_glrlm_GrayLevelVariance  wavelet-LLL_gldm_DependenceVariance | Delta-radiomic=-0.10086-1.14279*original_firstorder_Skewness  +0.69242*wavelet-HHL_glszm_GrayLevelNonUniformity-0.76156*wavelet-LLH_glrlm_GrayLevelVariance-0.83945* wavelet-LLL_gldm_DependenceVariance |
| Clinical | ER、HER2 | Clinical=-0.04205-1.38173*ER +1.36370*HER2 |
| Pre-radiomic+ Clinical | Wavelet-HHH_gldm_DependenceEntropy  wavelet-LHH_gldm_  SmallDependenceLowGrayLevelEmphasis  ER、HER2 | Pre-radiomic+Clinical=-0.07163+1.15278*wavelet-HHH_gldm_DependenceEntropy+0.96317*wavelet-LHH_gldm_ SmallDependenceLowGrayLevelEmphasis  -1.56327*ER+1.25292*HER2 |
| 1st-radiomic +Clinical | original_ngtdm_Coarseness  wavelet-HHH_glcm_Idmn  wavelet-LLH_glcm_ClusterProminence  ER、HER2 | 1st-radiomic+Clinical=-0.01748+0.56344*original_ngtdm_Coarseness-0.50850*wavelet-HHH_glcm_Idmn+0.93715*wavelet-LLH_glcm_ClusterProminence-  1.19668*ER+0.89853*HER2 |
| Delta-radiomic +Clinical | original_firstorder_Skewness  Wavelet-HHL_glszm_GrayLevelNonUniformity  wavelet-LLH_glrlm_GrayLevelVariance  wavelet-LLL_gldm_DependenceVariance  ER、HER2 | Delta-radiomic+Clinical=0.24293-1.53854*original_firstorder_Skewness  +1.01146*wavelet-HHL_glszm_GrayLevelNonUniformity  -0.80616* wavelet-LLH_glrlm_GrayLevelVariance  -1.24139*LLL_gldm_DependenceVariance-2.16803*ER+2.14384*HER2 |





**Figure S1** Lymph node and breast pathologic complete response (pCR) are illustrated.

**
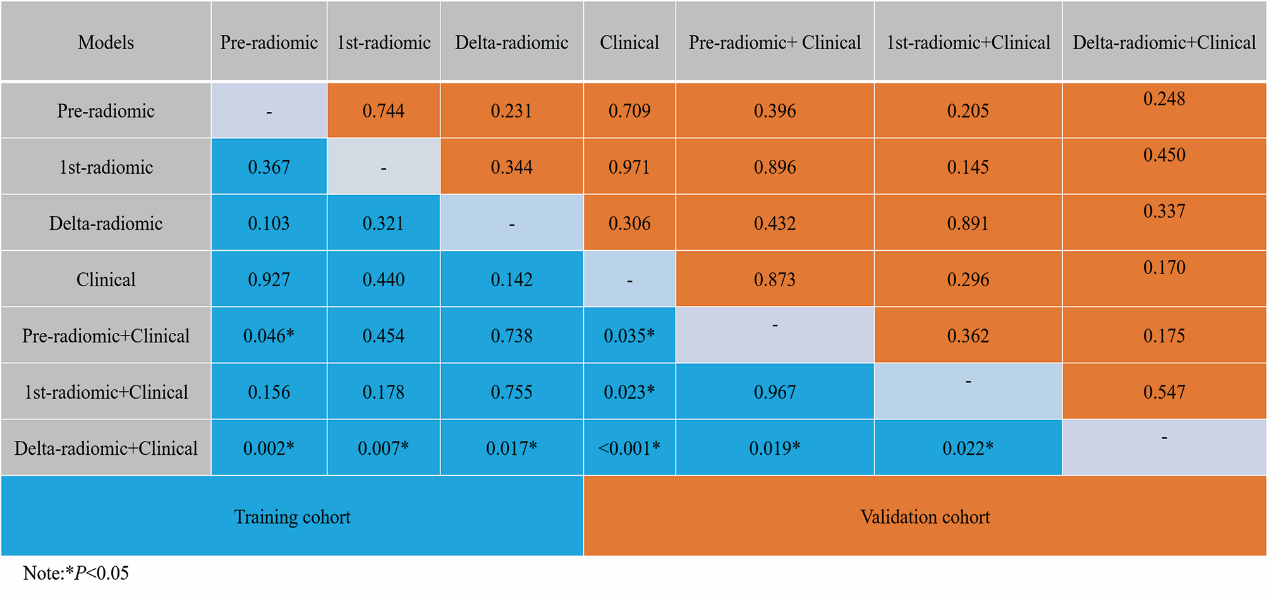
**

**Figure S2** Delong test for all models in the training and validation cohorts.
